# Informality and employment vulnerability: application in sellers with subsistence work

**DOI:** 10.11606/S1518-8787.2017051006864

**Published:** 2017-11-07

**Authors:** María Osley Garzón-Duque, María Doris Cardona-Arango, Fabio León Rodríguez-Ospina, Angela María Segura-Cardona

**Affiliations:** I Facultad de Medicina. Escuela de Graduados. Universidad CES. Medellín, Antioquia, Colombia; II Facultad de Medicina. Escuela de Graduados. Universidad CES. Medellín, Antioquia, Colombia; III Departamento de Ciencias Básicas. Facultad Nacional de Salud Pública. Universidad de Antioquia. Medellín, Antioquia, Colombia; IV Escuela de Graduados. Universidad CES. Medellín, Antioquia, Colombia

**Keywords:** Work, Unemployment, Working Conditions, Workplace, Occupational Health Social Vulnerability, Trabajo, Desempleo, Condiciones de Trabajo, Lugar de Trabajo, Salud Laboral, Vulnerabilidad Social

## Abstract

**OBJECTIVE:**

To describe the origin, evolution, and application of the concept of employment vulnerability in workers who subsist on street sales.

**METHODS:**

We have carried out an analysis of the literature in database in Spanish, Portuguese, and English, without restriction by country. This is a review of the gray literature of government reports, articles, and documents from Latin America and the Caribbean. We have analyzed information on the informal economy, social-employment vulnerability, and subsistence workers.

**RESULTS AND CONCLUSIONS:**

The concept of informal economy is dispersed and suggested as synonymous with employment vulnerability. As a polysemic term, it generates confusion and difficulty in identifying defined profiles of employment vulnerability in informal subsistence workers, who sell their products on the streets and sidewalks of cities. The lack of a clear concept and profile of employment vulnerability for this type of workers generates a restriction on defined actions to reduce employment vulnerability. The profiles could facilitate access to the acquisition of assets that support their structure of opportunities, facilitating and mediating in the passage from vulnerability to social mobility with opportunities. We propose as a concept of employment vulnerability for subsistence workers in the informal sector, the condition of those who must work by day to eat at night, who have little or no ownership of assets, and who have a minimum structure of opportunities to prevent, face, and resist the critical situations that occur daily, putting at risk their subsistence and that of the persons who are their responsibility, thus making the connection between social and employment vulnerability.

## RESUMEN

**OBJETIVO:**

Describir el origen, evolución y aplicación del concepto de vulnerabilidad laboral en trabajadores con empleos de subsistencia en las ventas callejeras.

**MÉTODOS:**

Análisis de la literatura en bases de datos en idioma español, portugués e inglés, sin restricción por país. Revisión de literatura gris de informes gubernamentales, artículos y documentos de América Latina y el Caribe. Se analizó información de economía informal, vulnerabilidad sociolaboral y trabajadores con empleos de subsistencia.

**RESULTADOS Y CONCLUSIONES:**

El concepto de economía informal es disperso y se sugiere como sinónimo de vulnerabilidad laboral. Como término polisémico, genera confusión y dificultad para identificar perfiles concretos de vulnerabilidad laboral en trabajadores informales con empleos de subsistencia, que venden sus productos en las calles y aceras de las ciudades. No disponer de un concepto claro y un perfil de vulnerabilidad laboral para este tipo de trabajadores genera restricción en acciones concretas, para reducir la vulnerabilidad laboral. Los perfiles podrían facilitar el acceso a la adquisición de activos que apoyen su estructura de oportunidades, facilitando y mediando en el paso de la vulnerabilidad a la movilidad social, con oportunidades. Se propone como concepto de vulnerabilidad laboral para trabajadores del sector informal con empleos de subsistencia, la condición de aquellos que deben trabajar de día para comer de noche, que presentan una escasa o inexistente posesión de activos y una mínima estructura de oportunidades para prevenir, hacer frente y resistir las situaciones críticas que se les presentan cotidianamente, poniendo en riesgo su subsistencia y la de las personas que tienen a su cargo, conectándose así vulnerabilidad social y laboral.

## INTRODUCTION

Informal economy and informal employment characterizes the predominant economic model^52^. According to the 2013 Labour Overview of the International Labour Organization (ILO)^42^, in Latin America the employed population in this sector of the economy is approximately 50%, which also mentions that in recent years the focus is on women and young persons.

Within the population of workers employed in the informal sector we can mention those who have informal jobs, which in turn have been classified and reclassified by the ILO since 1993^34^ ^,^ ^35^ ^,^ ^41^ ^,^ ^43^ ^,^ ^44^, without obtaining until the moment a consensus that allows clear comparisons between countries and the specific definition of how the groups of workers who exercise their profession making streets and sidewalks their place of work should be considered. They are referred to indiscriminately in information from official research studies and reports as workers of the informal sector, workers with informal jobs, with precarious jobs, with subsistence jobs, poor workers, and, more recently, as vulnerable working population, without yet having the necessary visibility for an adequate support. The considerations above make it difficult to define the concept of employment vulnerability as a major component of social vulnerability, as it relates to the assets and opportunities that could enable workers to anticipate, face, and resist situations that could affect them personally and their family.

The above situation reflects the need to define a concept of employment vulnerability that facilitates the methodological advance to identify conditions and characteristics of employment vulnerability in workers with subsistence jobs, especially sellers of products in the streets and sidewalks of cities. Generally using a concept that involves such a wide range of particularities makes it difficult to make decisions for the prioritization of actions at different decision levels.

All the above conditions and characteristics are part of a decent work, as postulated by the ILO^34^ ^,^ ^37^ ^,^ ^40^, and they make it more unattainable because if the concept is not specified, its measurement and application in the field will hardly materialize, leaving in a complex scenario the actions that may be well-intentioned, but which are scarcely directed to address employment vulnerability.

It should be understood that this term was driven by the exhaustion of the concept of poverty and by the search for new ways of analyzing and dimensioning a problem with impacts on public health, which implies both the description of the working poor and the identification of their ability or inability to foresee, face, and withstand potentially harmful or dangerous situations.

For the reasons explained above, this review aims to describe the origin, evolution, and application of the concept of employment vulnerability in informal workers with subsistence jobs from street sales.

## METHODS

We carried out a bibliometric study of critical narrative review in four databases, with review of articles, gray literature, government reports, and international agencies for Latin America and the Caribbean. The search procedure focused on the exploration of studies, articles, academic documents, and reports from governmental and non-governmental agencies (such as the World Health Organization/Pan American Health Organization (WHO/PAHO), ILO, United Nations Environment Program (UNEP), Latin American and Caribbean Demographic Center (CELADE), Economic Commission for Latin America and the Caribbean (ECLAC), among others), directly on their official pages, which referred to the context of informal economy, its references, uses, and applications in general, connection of the informal economy with employment vulnerability, and its characteristics and relations. Information on social vulnerability was sought as a broad context that includes employment vulnerability, focus, and practical applications in the ministerial pages and in the National Administrative Department of Statistics (DANE Colombia). Information that was not searched in the database was retrieved as gray literature using Google Scholar.

We consulted the scientific databases PubMed, SciELO (Brazil, Mexico, Spain, Argentina, Chile, and Colombia), Bireme, and LILACS for topics related to informal employment, subsistence jobs, and their relation with social and employment vulnerability, besides including general labor and social information for the informal sector.

The keywords selected by prior knowledge of the topics and for convenience for the topic of interest were: *«vulnerabilidade laboral», «vulnerabilidade social», «trabalho informal», «empregos de subsistência», «vulnerabilidade socioeconômica»,* and *«ambulantes de rua».* Initially, the terms were searched and, once all records were captured, the Boolean terms AND and OR were used to refine the search for all indexes with *«economia informal», «empregos precários», «laboral», «street workers»,* and *«social»*. The Table presents the search criteria and the Figure presents the criteria for withdrawing the documents. Articles were searched in Spanish, Portuguese, and English, without restriction by year. The search for government reports and international agencies was made for Latin America and the Caribbean.

**Table:** Summary of bibliographic revisions made in databases and in other literature, for the concept and use of informality and employment vulnerability, according to type of source and reference.

Type of source	Reference	Found	Selected	Topic 1	Topic 2	Topic 3
Databases	Scielo					
	Brazil	799	8	-	-	8
	Mexico	1	0	-	-	-
	Argentina	9		-	4	5
	Colombia	30	1	-	1	2
	Chile	0	0	-	-	-
	PubMed	1,180	9	-	2	5
	Bireme	11	8	1	1	6
	LILACS	12	10		3	7
	Subtotal	2,043	37			
Gray literature	CEPAL/CELADE, PNUMA	80	7	-	7	-
	ILO	50	21	11	10	-
	PAHO/WHO	10	3	1	2	-
	Official documents/Colombia	8	6	1	5	-
	Other documents/articles	21	14	5	7	2
	Subtotal	169	51			
Total		2,212				

ECLAC: Economic Commission for Latin America and the Caribbean; CELADE: Latin American Center for Population Studies; UNEP: United Nations Environment Programme; ILO: International Labour Organization; PAHO/WHO: Pan American Health Organization/World Health Organization

**Figure:**
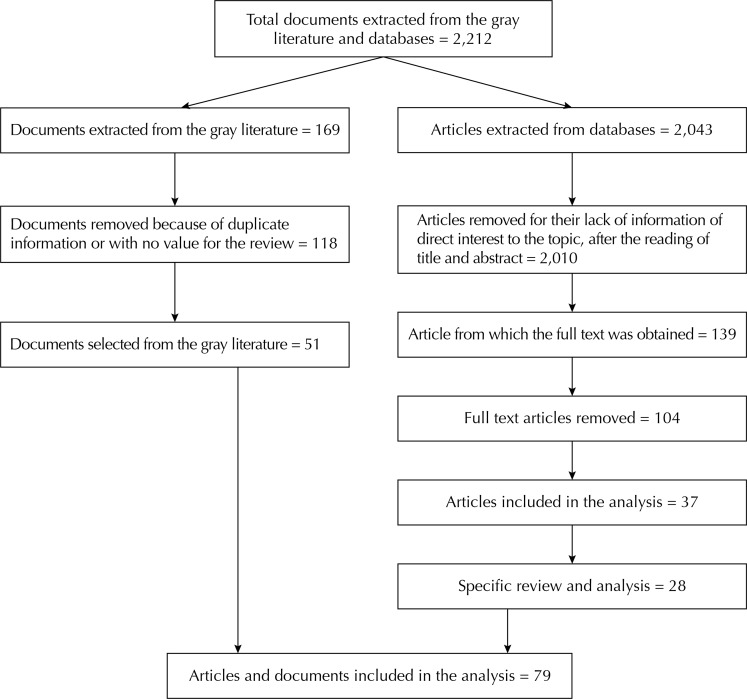
Document debugging tree of informality and employment vulnerability.

With the bibliographic review classified within the gray literature, 169 documents, official reports, articles, among others were identified and 51 of them met the selection criteria (Table).

In the scientific databases, 2,043 articles were retrieved and after refining the specific thematic approach with the removal of duplicate articles, and taking into account the criteria described previously, 37 articles were selected. In all of them, a review of all abstracts and introduction was carried out to verify if they contained elements that would guide the topic of employment vulnerability related to subsistence work.

Finally, the review and specific analysis of subsistence work, as the last category of interest, was performed with 28 of the 37 selected articles (Figure).

One of the researchers carried out a review of abstracts and other researchers carried out a second review to verify the classification of articles according to their relevance in the approach to the topic. We also verified the pertinence of the gray literature documents that were circumscribed to the countries of Latin America, and the actuality of the documents and articles, considering the changes in the different ways of valuing informality and employment vulnerability. We also took into account that the scientific articles included the previously defined search terms. The central topic was subdivided into three subtopics: (i) informal economy, context, and characteristics, (ii) socio-employment vulnerability, and (iii) subsistence workers.

## RESULTS

The type of work and the economic model are elements that condition the poorest populations, and subsistence jobs contribute to the configuration of poverty^8^. The population growth and the economic model facilitated the individualization of responsibilities^10^ ^,^ ^24^ which were previously of the companies or Government, a change that led to a growth of 3.9% per year of the informal economy between 1990 and 1998, while the formal sector growth was 2.1% in the region^33^ of Latin America; moreover, negative effects affected the health of vulnerable populations because of the decrease in social spending in many countries^45^.

According to official reports and scientific evidence registered in Latin America and Europe^8,22,27–29,46,47,62^, the health of the poor and vulnerable populations in the world could be improved by taking into account that the social conditions in which persons live and work contribute to their disease and health conditions^47^.

The ILO in 2013^42^ mentioned that three in every 10 Latin American and Caribbean workers do not have access to any type of social protection coverage, and young adults represent approximately 50% of the unemployed persons in urban areas. Women continue to be affected by unemployment and the rate of informality (47.7%) persists for non-agricultural workers, a situation which is recorded in the ILO 2011 report on world employment trends^39^. The ILO^36^ ^,^ ^38^ and the Ministry of Health of Colombia^13^ ^,^ ^a^ mention that vulnerable working populations are considered as those that have a subsistence work and with precarious employment because of their low education level, lack of economic resources, and fragile ties with social security systems^42^. These populations are also at social, occupational, and community risk, hindering the implementation of some of the actions indicated by the Regional Decent Work Plan^26^ ^,^ ^48^ ^,^ ^a^ ^,^ ^b^, especially when the meaning of informal worker is difficult to define and direct^56^.

An indicator of poor workers is that of “vulnerable employment”^34^, which consists of self-employed and unpaid family workers. They, because of their vulnerability, often have informal work agreements. Employment vulnerability is also often characterized by low wages and poor working conditions, which may undermine the fundamental rights of workers^26^. According to ILO in 2002, the employment rights acquired for workers in the informal sector of the economy are infringed.

In Colombia, the 2012-2021 Decennial Public Health Plan (PDSP)^a^ includes the vulnerable working population, but it has poorly defined guidelines for “street vendors” with informal employment, who constitute an alternative for those who cannot work in the formal sector and have low education level^c^.

To measure vulnerability, the National Planning Department (DNP) in Colombia^d^ has an indirect indicator and mechanisms of focus for social spending, in the population that includes workers with needs of employment subsidy. Social health care is designed for a category of persons in poverty and vulnerability, which can be reduced or mitigated with risk management strategies^d^.

On the other hand, vulnerability can be seen as a process of conditions that are accumulated and vary in time and space and as a function of the subsistence mechanisms of individuals and households and the investments they can make in the medium and long term in the reproduction of their social system^15^. For the World Bank^e^, those who receive low earnings because they are employed in the informal sector of the economy are also prone to suffer from preventable diseases and face a high risk of social exclusion.

The concept of informal economy, from the institutionalist perspective^23^ ^,^ ^f^ ^,^ ^g^ favors the individualization of responsibilities, and the worker is responsible for his or her protection against the risk factors of accidents or diseases^h^. The Ministry of Social Security has a role to monitor and configure the current situation in occupational health for the informal sector of the economy^30^, in which conditions of precariousness and employment vulnerability can be observed, which could be modified if profiles of vulnerability were identified in the workers.

The employment statistics of the National Administrative Department of Statistics (DANE) operatively define urban informal employment, including self-employed workers, other than independent professionals and technicians^f^ ^,^ ^d^ (Box 1).

**Box 1:** Definitions of informal economy, guidelines for measurement, and classification of employment vulnerability within labor informality.

Country/ Institution	Definition^a^	Categories or topics to be measured^b^	Classification^c^	Valuation^d^
ILO, 1993	We have *workers who cannot be classified according to the employment situation*. *Subsistence workers* appear within the statistical treatment of particular groups, literal S, thus: s) Subsistence workers: workers with independent employment, for whom the product of their work is an important source of their subsistence. (see paragraph 7)	Informal employment Subsistence workers	Subsistence workers are defined, but it is not defined in detail whether street vendors of products and services are included in this group.	Broad definition and little defined.
ILO, 1993	Decent work: Defined by ILO as one that, in addition to being productive, can be chosen freely and have fair compensation according to the work done, allows the worker and the persons who depend on him or her to live in a dignified way, not simply survive. It is the work that is carried out under the premise of respect and fulfillment of the rights and dignity to the workers, free of exploitation, which allows the worker to provide his or her services to the population, a situation in which will be seen simply as a factor of production.	1. Tends to improve the quality of life of work environments; 2. Contributes to policies and legislation of formulation; 3. Promotes workers’ health; and 4. Offers integral health services to workers.	It defines what will be considered a decent job, but the sector of the economy is not defined where these unfavorable conditions could be present, nor the type of jobs and workers to which the categories can be applied.	Broad definition and little defined.
ILO, 2003	The conceptual framework elaborated by ILO is explained, noting the conceptual relationship between informal sector and informal employment, in addition to the concepts of underground and illegal production (as defined in the 1993 SNA). It is mentioned the preference to manage independently the concept of informal sector and informal economy for statistical purposes; although the concept of unprotected employment is proposed to replace informal employment, the latter will continue to be considered.	Informal, job–based employment. Informal sector based in the company. Informal work. Employment in the informal sector. Informal employment.	The conference advises to keep the concepts of employment in the informal sector and informal employment separated.	Broad, little defined in relation to the occupations of street vendors.
ILO, 2008	In this classification of occupations no allusion is made to the definitions of occupations of the formal or informal sector of the economy; the names and their codes by occupation are simply listed. Within the nine large groups, informal street workers could be included implicitly or explicitly in 5 and 9, as described in the column of categories or topics to be measured.	5. Service workers and sellers of trades and markets, which include: sellers (52), street and market vendors (521), merchants and store and warehouse sellers (522), hawkers and sellers of edible products (523), and other vendors (524). 9. Elementary occupations: hawkers of services and related workers, and street vendors of services and related workers in this subgroup, and hawkers (excluding food vendors)^49^.	It defines the occupations and includes in them those that comprise the informal street workers and the subsistence workers are part of the agricultural workers.	Classification defined by occupation and indefinite by the sector of the economy.
2015, Spain	The conceptual discussion on informal employment is based on documentation from the ILO. Since the beginning of the administration of the term «informal sector» in the early 1970s, it has gone through several stages, going from being considered a phenomenon reduced to companies operating in the informal sector to the understanding that informality transcends the informal sector and which could also be found in other economic activities. The term «informal economy» has responded to the goal of directing a better understanding of the phenomenon.	Informal employment. Informal sector. Informal economy. Informal work. Precarious employment. Definition, measurement, and classification of informal employment in the countries of South America.	One could not be defined, because of the observation of a disparity in the definitions used; although in many countries informal employment is defined at least as non-contract workers, since there are different classifications, the information found is heterogeneous, with little standardized records, thus no regional comparison can be made. The definition of the International Labor Organization is not sufficient to analyze the context studied. This document concludes that separation of protected and unprotected persons should be incorporated into the definition of informal employment.	Broad definition, not defined to a type of workers.
2011, Chile	It groups in one category certain economic activities related to low productivity and poverty. The analysis of informal employment was associated with subsistence activities for persons with jobs in the marginal sectors of the economy; from this point of view, it is considered that the informal sector is the result of a surplus of labor per job. More recently, Alejandro Portes’ view of informal work, unlike that of the International Labor Organization (ILO), understands that informality should not necessarily be linked to poverty, and that informality should not necessarily be associated with urban sectors (it should be seen as late capitalism, flexibilization of labor laws).	Informal work. Informal sector. Informality. Activities not regulated by the Government. Consequences of the use of the concept.	Definition and consideration of informality of a deinstitutionalized view; however, it continues to be unspecific and states the difficulties to define it, since it considers that there is a wide range of workers who are almost informal and almost formal.	Broad definition, not defined to a type of workers.

OIT: International Labour Organization; SNA: System of National Accounts

^a^ Definition: according to what is defined by the governmental entities of each country and agencies such as ILO, WHO/PAHO, UNDP, among others.

^b^ What aspects of informality and employment vulnerability could be measured according to official definitions.

^c^ What limitations are identified in the definition to classify workers and identify the elements that are excluded.

^d^ Valuation: confused, limited, incomplete, broad. Confusing when official documents are not accurate in relation to the definition, classification, or aspects that should be measured; limited, where there is a definition and the criteria are precise, but the elements needed to be considered are missing; incomplete, when the definition is more advanced, but important elements still need to be incorporated; broad, but incomplete, when there is a good definition and the aspects that will be measured include almost the entire spectrum of informal workers who are considered as vulnerable, but there are some elements that are not included or that are not clearly defined.

The concept of vulnerability was used for the analysis of environmental risks and hazards^9^ ^,^ ^15^ ^,^ ^i^, as well as for demographic^e^ ^,^ ^j^ and economic conditions^k^. In addition, it was resumed and adjusted to be incorporated into the social analyses, deriving the concept of social vulnerability as the inability to foresee, face, and resist a harmful situation^l^ ^,^ ^m^, which is a concept for the analysis of poverty in a more integral way. Some authors^n^ ^,^ ^o^ mention that this concept will identify the shortcomings and propose to focus attention on the assets that can facilitate the responsiveness of communities, families, and persons to adverse situations, potentiating changes from what they have, rather than from what they need^31^ ^,^ ^k^ ^,^ ^m^ ^,^ ^o^ ^,^ ^p^.

The assets for the analysis of social vulnerability are physical (financial and physical, as in the case of housing), human (work, health, and education), and social (social networks) capitals. These, on the other hand, are generated especially by the Government, market, and community^m^ – which is one of its forms of materialization –, and they can potentiate the assets or exacerbate situations of poverty, shortages, and danger.

The study of social vulnerability for different countries of the American continent mentions that work is of great importance in achieving social balance. This may have been one of the reasons why the ILO, at the end of the 1990s, requested the analysis of the social vulnerability of Uruguay, including employment^n^, perhaps in search of other tools and arguments to address the growing and irresistible labor informality in the continent. This process gives way to the emergence of a model to measure social vulnerability, which, in addition to valuing deficiencies, aims to identify the assets of the poor, as well as their structure of opportunities to foresee, face, and resist situations of threat or danger; this model is designated as the Model of Assets, Vulnerability, and Structure of Opportunities (AVEO)^25^.

Employment vulnerability is included in social vulnerability, thus making the connection with informal economy; this is reflected in the report and analysis of the ILO^40^ ^,^ ^48^ ^,^ ^52^, international agencies^49^ ^,^ ^p^, health and labor authorities^30^ ^,^ ^a^, and in the studies carried out for the population with informal jobs in Colombia^51^, Brazil^17^ ^,^ ^24^ ^,^ ^53^, Mexico^21^, Chile^7^, and Argentina^13^ ^,^ ^18^ ^,^ ^59^.

Social vulnerability seeks to identify conditions that go beyond material poverty to contribute with elements for prioritization and delivery of subsidies^31^ ^,^ ^m^ ^,^ ^n^, showing, at least in the concept, an advance in relation to the poverty approach that was inefficient to reverse the condition of poverty in persons, families, and communities.

In the studies and documents analyzed, a scattered and confused use of the concept of employment vulnerability was observed, depending on the country, the agency reporting the official reports, and the type of workers referred to in them.

The concept goes from a marginal use, applied to workers in the formal sector, called precarious jobs^2^ ^,^ ^4^ ^,^ ^11^ ^,^ ^61^ to the application to workers with different skills within the informal sector of the economy, as mentioned in the reports of ILO and studies from different countries, finding that the common factor is workers without affiliation to general social security systems, with low education levels, and scarce economic gains^q^.

For Pizarro^k^, what makes the conditions of employment vulnerability more evident are: the accentuation of productive heterogeneity that affects the occupation, the segmentation of the work and greater precariousness, employment deregulation (or flexibilization) without unemployment insurance, the reduction of the quantitative weight of unions, and the sustained growth of labor informality, especially for city workers.

The United Nations Development Programme of the Economic Commission for Latin America and the Caribbean (UNDP-ECLAC)^54^ considers that vulnerability and poverty are converging phenomena, describing, among other things, that “the current social scenario simultaneously records an increasing uncertainty regarding work as the main way of building the future of persons and their families”^54^. Johnson, in the ILO report^q^ in 2010, when mentioning the report “world employment trends”, says that by increasing vulnerable employment in the world by more than 100 million, poverty is also increased, and he notes that self-employed workers and family workers are considered as vulnerable, because difficulties with social security, corporate representation, and lack of decent work are more likely in them^q^.

Box 2 reflects an extensive and inaccurate use of the concept of employment vulnerability in subsistence workers. Diverse topics are described, such as employment vulnerability for work-related accidents in the formal sector of the economy^62^ and globalization and its impact on the vulnerability of workers by the flexibilization of job relations^6^. They also refer to the relation of work and health in the migrant population^1^, considered as a population with precarious employment.

**Box 2:** Characteristics of the articles included in the review that were identified with the terms of employment vulnerability, social vulnerability, informal employment, subsistence work, and street vendors.

Author	Country of study	Type of study	Group	Does it define the concept of employment vulnerability	Valuation^*^
Smith PM, Saunders R, Lifshen M, Black O, Lay M, et al.	Spain (includes European countries and the USA)	Systematic review and descriptive study	Migrant workers	Not exactly; it describes the development of measurement in occupational health, safety, and vulnerability for work accidents for the population of migrants from the formal or informal sector of the economy.	Specific for its application in work accidents for migrant workers.
Hernández P, Zetina A, Tapia M, et al.	Mexico, Mexico City	Descriptive	426 female sellers	It does not refer to the concept of employment vulnerability; it mentions female workers in the informal economy, such as female street vendors in four districts of Mexico City.	It does not define.
Ahonen EQ, Benavides FG, Benach J	Spain (includes European countries and the USA)	Review	Agriculture, construction, daily jobs, taxi drivers, other	Not exactly; the development of measurement of occupational health, safety, and vulnerability in the immigrant population is described.	Non-specific.
Sánchez AIM, Bertolozzi MR	Brazil	Systematic review	Health users, patients, and their families	This document reviews the concept of vulnerability to contribute to the support of health care in Brazilian cities.	Confusing.
Cross C	Argentina	Qualitative	Women who participated in a construction program.	It refers to the concept of employment vulnerability applied to persons with low employability, particularly in working women who participated in a social construction program in which recycling plants were built in a landfill in Buenos Aires, Argentina.	Broad, social vulnerability in persons with low employability
Tasso AT, Zurita C	Argentina	Qualitative	Workers who move places for agricultural activities	It is located in the concept of social vulnerability and decent work to value the conditions of migrant workers in agricultural tasks by seasons, jobs that are recognized as “swallow” jobs, since workers travel from Santiago del Estero to different states of Argentina.	Broad, social vulnerability in persons with agricultural jobs called “swallow” – decent work.
González LM, Ortecho M, Molinatti F	Argentina	Review	Documents and small neighboring areas of Argentina	It summarizes the notion of social vulnerability in the Latin America, but it locates and defines its analysis in the concept of social mobility in small neighboring areas. There is no direct reference to the working population.	Broad, social vulnerability in small neighboring areas of Argentina
Goldberg A	Argentina	Literature review and quantitative	Documents	Description of contexts of social vulnerability accompanied by health risk situations of illegal Bolivian migrants with tuberculosis who also work in non-formal textile workshops in Buenos Aires.	Broad, social vulnerability in workers
Palacios-Pérez AT, Sierra-Torres CH	Colombia – Popayán	Quantitative descriptive, research, and audiometry	186 hawkers of Popayán	The terms informal economy, informal sector, hawkers, and precarious employment by the economic model were mentioned; the concept of employment vulnerability is not mentioned. Topics related to prevalence and risk factors associated with communicative changes are also addressed.	Non-specific, it is not mentioned directly.
United Nations Development Programme (PNUD)	New York	Quantitative	Poor persons, workers, women, entire communities of the different countries in the continent	It covers a holistic approach to vulnerability, which includes resilience. The AVEO approach is assumed although it is not mentioned directly, since it talks about giving assets and capabilities to the vulnerable population in social and labor terms, to prevent, face, and recover from unfavorable situations, and to overcome the concept of poverty. It is questioned who are those vulnerable, to whom they are vulnerable, and why the populations are vulnerable, differentiating and considering employment as a part of social vulnerability.	Broad, social vulnerability in the general population; includes the working population.
Andrade MI, Laporta P	Argentina	Quantitative	Agricultural producers	Although the title announces the concept of social vulnerability of the agricultural producers of an Argentine region, this concept is not clearly defined in it; however, there is a permanent reference to the concept of social theory of risk and little employment vulnerability.	Broad, social vulnerability in agricultural producers; non-specific for labor.
Moyano DE, Guevara RC, Lizana JL	Chile – Maule region	Mixed	258 hawkers of the Maule region in Chile	It does not mention the concept of employment vulnerability directly; it refers to the term informal economy workers, which include self-employed, those engaged in subsistence activities, and within these it focuses on street vendors.	Non-specific – informal economy workers – self-employed – subsistence activities.
Bachiller S	Argentina	Qualitative – ethnographic	Informal waste pickers	Informal pickers are referred to as subsistence workers and it is described that an analysis of the notion of alienation will be made in the analysis of employment precariousness that affects the pickers. Pickers in the waste dump of Comodoro Rivadavia of Patagonia in Argentina.	Confusing – it refers to subsistence workers and precarious employment.
Vargas MC, Aparicio AT, Alanís JC	Mexico	Review	Documents that show the topic in question	Meanings of the concepts of socioeconomic vulnerability, environmental justice, and spatial justice, in their relations with social risks and disasters. It does not refer to specific populations. The concept of socioeconomic vulnerability is administered as a key to understanding the causes of disasters.	Broad – socioeconomic vulnerability, environmental justice, space justice.
Ospina JM, Manrique FG, Ariza NE	Colombia – Boyacá	Quantitative – cross-sectional, descriptive	1,410 potato growers from the central state of Boyacá	Health, environment, and work in vulnerable populations: potato growers in central Boyacá. In relation to the concept of employment vulnerability, the article mentions that the informal sector is a vulnerable population; in general workers have low paid employment, long hours of work, and mediocre and unregulated working conditions, conditions that generate bad life conditions.	Broad – vulnerable populations.
Salas MM, Oliveira O	Mexico	Mixed	Young persons and adolescents	It studies how the crisis has particularly aggravated the employment vulnerability of adolescent workers, from the more frequent processes of informalization, precarization, and lack of labor protection.	Confusing – vulnerability, precarious employment with subsistence work, unprotected work, etc.
Pamplona JB	Brazil	Quantitative	Street vendors of São Paulo	It does not define the concept of vulnerability in the text. It mentions as an important point the decrease in street vending in São Paulo, which went from 133,000 in 2004 to 100,000 in 2009, and this is considered as a consequence of an improvement in employment. It is described as the sale of illegal products and that can cause problems of mobility and illegality. It does not define a concept of vulnerability; it mentions it with informal workers.	Non-specific – informal economy workers
Roa JCG, García-Suaza A, Rodríguez-Acosta M	Colombia	Quantitative	Home Survey Information GEIH-DANE 2010	It does not define the concept of vulnerability; it analyzes the process of implementing the concept of informality in the analysis of the Colombian labor market, centered on the workplace beyond the size of the place of work. These conditions show the questioning of public policies that have occurred for the administration of labor informality in Colombia.	Non-specific – informality.
Giatti L, Barreto SM, César CC	Brazil	Quantitative	National survey data – a sample of men aged between 15 and 64 years	It presents the concept of precarious employment; it does not mention employment vulnerability. However, an analysis of unprotected employment in the informal and health sector for workers with temporary contracts in Brazil between 1998 and 2003 for one of the eight metropolitan regions of Brazil is presented. It is described lack of social security, unemployment, and unprotected employment (heterogeneous groups).	Non-specific – informality – precarious employment.
Massi MF	Argentina	Quantitative	Mixed – microdata from research	Dimensions of precarious employment in Argentina: a broad set of working conditions is mentioned here; it is described that it is created linked to the instability in hiring and exclusion from the labor market. It explains that there are no ‘precarious’ and ‘not precarious’ jobs; there are smaller or greater degrees of precariousness in the different segments of the production structure, since in general, all have characteristics of precariousness.	Non-specific – it addresses precarious employment for workers in different sectors.
Gómez PI, Castillo AI, Basquez SA, Castro OA, Lara EH	Colombia – Cartagena	Quantitative	584 informal stationary sellers of the city market (Bazurto)	It mentions that the sector of informal employment brings together a vulnerable population, which is poorly remunerated and with long and strenuous hours, and in general, the precarious working conditions affect the living and health conditions of workers^20^.	Confusing – it mixes employment vulnerability and precarious employment indistinctly.
Garzon-Duque MO, Gómez-Arias RD, Rodriguez-Ospina FL	Colombia – Medellín	Quantitative	422 informal street workers on the streets and sidewalks of the Medelin center	It defines as vulnerable working population those who are at social, occupational, and community risk; it alludes to the regional decent work plan, to subsistence jobs within the informal economy. It defines that they are sellers who work during the day to eat at night.	Specific – but not defined for sellers.
Sotelo-Suárez NR, Arcentáles JLQ, Montilla CPM, López-Sánchez PA	Colombia – Bogotá	Quantitative	3,936 working women in informal economy in different areas	It does not define the concept. It mentions conditions of precariousness of women with low education level, gains below the legal minimum wage, with long working hours, scarce time to carry out activities of leisure and free time; it mentions that 75% of the women are heads of households.	Non-specific – it refers to the concept of precarious employment to define employment vulnerability of the women under study.
Ballesteros LV, Arango YLL, Urrego YMC	Colombia – Rural Area of Medelin	Quantitative, cross-sectional, descriptive	100 informal pickers of five neighborhoods of Medelin in 2008	It designates workers as informal environmental pickers; however, informal picker, vulnerable worker, or subsistence worker is not clearly defined. However, it describes that their informal conditions expose them to working conditions that affect their health and that of their families. Their health and work conditions are precarious. The biological, ergonomic, and social risks to which they are exposed and lack of social security constitute their precariousness. Non-unionized workers are a large majority in relation to unionized workers, more women than men, mostly single, and with low education level.	Non-specific – it refers to the concept of precarious employment but not defined, which mentions precarious employment for environmental pickers, both unionized and non-unionized. It insinuates more than its precariousness, the employment vulnerability of non-unionized workers.
Carvalho Junior LCS, Ramos EMC, Toledo AC, Ceccato ADF, Macchione M, Braga ALF, et al.	Brazil – West of the State of São Paulo	Quantitative – longitudinal descriptive	With 44 cane cutters, from whom data were collected in two moments in 2010	Workers in conditions of precarious employment in rural areas such as migrants from poor regions of Brazil, being on average 25 years old, with five years or less of education. The work they perform requires from them great physical effort, and the payment occurs by the quantity cut; they are exposed to polluted air and the sun, make too much physical effort, and do not consume water, situations that affect their physical and mental health. In general, with good perception of their state of physical and mental health.	Non-specific – it mentions precarious working conditions but does not define the concept for these cane cutters, nor the type of contract they have with the company where they work.
Teixeira JRB, Boery EN, Casotti CA, Araújo TMd, Pereira R, Ribeiro ÍJS, et al.	Brazil – Jequié, Bahia State	Quantitative, cross-sectional, descriptive	Made with 400 motorcycle taxi drivers, published in 2015	It wanted to explore their perception of quality of life and see if the control over their own work had repercussions on this perception. It mentions that motorcycle taxi is a labor activity arising from informality, in which the drivers undergo diverse conditions to increase their gains, although they imply malaise, damages, or health problems. It mentions the precariousness of the work of motorcycle taxi drivers.	Confusing – motorcycle taxi drivers are referred to as informal workers, subject to the violation of their health conditions, the precariousness of this employment, but it is not clearly defined.
Cavalcante-Nóbrega LP, Mello AF, Maciel MR, Cividanes GC, Fossaluza V, Mari JJ, et al.	Brazil – São Paulo	Quantitative, cross-sectional, descriptive	With 79 mothers of one sample per convenience	Mothers of 7 to 14 year old boys who work in the streets of São Paulo. Mothers live in a context of poverty (up to 6 persons in a house), of domestic violence, with mental health problems that affect the children. Only half of the mothers were paid for work, with a regular perception of their quality of life and an average of 4 children.	Non-specific – It does not mention at any point that the work of the children in the street is precarious, vulnerable, informal, or of subsistence.
Assunção AÁ, Silva LS	Brazil – metropolitan region of Belo Horizonte, Minas Gerais.	Quantitative, cross-sectional	With 1,607 workers	It explores the prevalence of mental disorders in bus drivers and ticket collectors to see if the traffic conditions or bus conditions were associated with the disorders. It was observed that urban collective transportation workers are frequently victims of precarious work conditions, which trigger health problems; however, no description is given in the article of what are the reasons, for the specific case, for precarious employment and being vulnerable to psychic suffering.	Non-specific – it mentions that workers are victims of precarious working conditions, but it does not define their concept, being them contract workers.

AVEO: Assets, Vulnerability and Structure of Opportunities; GEIH-DANE: Large Integrated Home Survey - National Administrative Department of Statistics

^*^ Valuation: confused, limited, incomplete, broad. Confusing when official documents are not accurate in relation to the definition, classification, or aspects that should be measured; limited, where there is a definition and the criteria are precise, but the elements needed to be considered are missing; incomplete, when the definition is more advanced, but important elements still need to be incorporated; broad, but incomplete, when there is a good definition and the aspects that will be measured include almost the entire spectrum of informal workers who are considered as vulnerable, but there are some elements that are not included or that are not clearly defined.

In Mexico, a study mentions the needs of female street vendors to take care of their children^21^, and another study analyzes the impact of the crisis on employment vulnerability, informality, and precarious employment in young persons^56^.

Argentina studies social vulnerability and job creation for populations defined as vulnerable because of their low employability, as well as the labor migration characteristics in Santiago del Estero, considering the workers denominated as “swallow”, and in what way this work meets the characteristics of decent work^59^. Reviews were also observed that refer to the general population with characteristics of social vulnerability in Latin America, social mobility in neighboring areas^20^, the dimensions of precarious employment^13^, the informal sector, and unregistered labor^r^, subsistence and alienating jobs, in the analysis of precarious employment in waste pickers^5^, situations of health risk in Bolivian migrants with tuberculosis who work and live in clandestine textile workshops in Buenos Aires^18^, among others.

Chile has a study with 258 street vendors from the informal economy in subsistence activities for the Maula region^32^. Brazil has a study that shows the decrease of street sellers in the country between 2004 and 2009^53^. There is also a study in Brazil which has approached motorcycle taxi drivers^60^ and their perception of health and unprotected employment in the short term, without defining the concept of employment vulnerability, using precariousness in informality as reference for this analysis^17^.

Other studies in Brazil^11^ present the perception of quality of life for cane cutters, motorcycle taxi drivers^60^, and mothers whose children work in the streets of São Paulo^12^, designating them as work with characteristics of precarious employment, whether they are contract workers or informal workers. Another study has also addressed the prevalence of mental disorders in bus drivers and ticket collectors^4^, describing that their employment also presents characteristics of precarious employment; however, it does not refer to subsistence or vulnerable jobs.

Colombia has specific analyses for informal workers with subsistence jobs who sell their products on the streets and sidewalks of cities; they are generally defined as vulnerable working population, as suggested by the regional decent work plan^48^. The studies were carried out between 2004 and 2013, including agricultural workers, workers in market squares, and street vendors, and they characterize the sociodemographic and economic conditions, level of affiliation to the health system, perception of some health conditions of these workers, among others, with studies carried out in Popayán^51^, Boyacá^50^, Cartagena^19^, Medelin^16^, Bogotá^58^, etc.

On the other hand, there is a national diagnosis of health and work conditions, in which close to 20,000 workers of the informal economy participated in 20 states^30^.

There are also studies such as the 100 environmental pickers in rural Medelin^26^, designated as having precarious and vulnerable jobs; however, the concept of employment vulnerability for these populations was not defined.

The description of labor informality in Colombia is based on information from the Large Integrated Home Survey – National Administrative Department of Statistics (GEIH-DANE 2010)^s^, being more difficult to specify a concept of employment vulnerability applied to a specific group and which can facilitate the targeting of actions at the national and regional level.

## DISCUSSION

The concept, use, and application of the term informal economy is as broad and diverse as the population to which it applies, and although it shares general characteristics, they cannot be unified in the particular, since they include workers that are considered as formal being in the informal economy, and workers who are in the formal economy being considered as informal^55^, in addition to many other derivations given by the job classifications of ILO^35^ ^,^ ^44^.

On the other hand, heterogeneity is present in the use of the term precarious employment, because for some countries such as Brazil the few field studies with informal workers or with precarious jobs is more related to the formal sector and workers with work contracts^4^ ^,^ ^11^ ^,^ ^55^ ^,^ ^60^ than to workers with subsistence jobs on the streets and sidewalks of cities.

The conceptual dispersion and the implications of the scarce differentiation between subsistence jobs, precarious employment, work in informal jobs, or the informal sector are still unclear.

The systematic reviews^55^ mention that informal employment in the countries of Latin America cannot be compared, since heterogeneous and poorly defined concepts are assumed which leave a nebulous area in which workers with subsistence jobs can be made invisible by the ILO classifications^t^.

We have also observed that each country, via targeting^30^ ^,^ ^g^ ^,^ ^h^, seeks to advance actions to improve the living and working conditions of those who have this type of jobs. This could be the case with street vendors.

According to the ILO classification (2008)^43^, agricultural workers were considered to be subsistence workers, but street vendors were not considered in the same way, which were classified as self-employed workers^43^, without indicating that this condition causes them to work by day to eat at night in a subsistence job.

Although the concept of social and employment vulnerability has been incorporated for some time in Colombia, subsidies aimed at overcoming poverty continue to be in force.

This concept of the different mechanisms of focus cannot be erased, disappearing the essence of the concept “social vulnerability” proposed by Moser^31^, and adjusted by Kaztman and Filgueira^15^ ^,^ ^n^ ^,^ ^o^ who propose seeing the assets of individuals, families, and communities as capitals.

Within these capitals, work is included as a starting point in overcoming deficiencies, and it was observed that, accompanied by a structure of opportunities, they allow the worker, the family, and the community to predict situations of potential loss, facing and resisting them.

In this sense, the perspectives could point to the overcoming of vulnerability, and the Government, market, and community ensure that they are available^61^ ^,^ ^p^.

Work can either boost assets or exacerbate situations of poverty, shortage, and danger, which is why it is not an issue of simply identifying the assets that workers may have.

In order for working populations to overcome adversities, they need a structure of opportunities that maximize both their acquisition and their effective materialization with decent, free, and protected work.

The informal worker designated as vulnerable with subsistence work could present low education level, difficulty accessing health systems, and weak ties with the labor market^34^; however, specific information is still scarce for different groups of vulnerable workers.

It is difficult to retrieve information that shows the defined profiles of employment vulnerability for those who make the streets and sidewalks of the cities their place of work with subsistence jobs, hindering both the application of the concept and its implementation in the field, and leaving particular situations in an area of uncertainty that must be addressed in a special way. With the above, social and work disadvantage conditions were evidenced in Latin America, particularly in Colombia^3,9,16–18,24,32,50,51,58,r^, where despite the evidence, these conditions are not taken into account for the reconfiguration of their social and labor peculiarities. This condition can lead to a greater deterioration of the physical and mental health of workers, accompanied by economic, social, and family consequences, making it more difficult to overcome employment vulnerability in terms of weakness to mobilize assets, use them, and the scarce or lack of opportunities to overcome poverty.

After reviewing and analyzing the concepts of informality, social-employment vulnerability and precarious employment, it can be said that there is little scientific evidence to define a concept that fits workers with subsistence jobs, understanding that within them we have those who make the streets and sidewalks of cities their place of work. A concept is needed to define the profile of precarious employment to move towards overcoming this condition, giving way to the fulfillment of the assumptions of the regional decent work program of ILO^34^ ^,^ ^37^ ^,^ ^40^.

Thus, it is evident the need for greater conceptual clarity that facilitates the methodological application for the analysis with this type of population.

As a result of this review, we propose that informal street workers are those who, having a subsistence job, work during the say to eat at night, and whose vulnerability exists because of their scarce or nonexistent possession of assets and a minimum structure of opportunities to prevent, face, and resist the critical situations that appear daily, endangering their subsistence and that of the persons they are responsible for, thus making the connection between social and employment vulnerability.

## Footnotes

a Ministerio de Salud y Protección Social (COL). Plan Decenal de Salud Pública, PDSP, 2012–2021. Bogotá (DC); 2013 [cited 2014 Aug 26]. Available from: https://www.minsalud.gov.co/sites/rid/Lists/BibliotecaDigital/RIDE/VS/ED/PSP/PDSP.pdf

b Organización Internacional del Trabajo. Informe anual sobre los derechos de los trabajadores. Trabajar en el sector informal: una oportunidad, un riesgo, un reto: dossier realizado para la Comisión Mundial del Trabajo por la agencia InfoSud-Belgique. Capítulo 3: condiciones de trabajo contrarias a las normas. June 2002.

c López H. Indicadores de precariedad del empleo urbano [conferencia]. In: Primer seminario Colombia Informal; 2006; Alcaldía-de-Medellín, Colombia.

d Departamento Nacional de Planeación (COL), Dirección de Desarrollo Social, Grupo de Calidad de Vida. Mecanismos de focalización: cuatro estudios de caso. Bogotá (DC); 2007 [cited 2015 Apr 1]. Available from: https://colaboracion.dnp.gov.co/CDT/Desarrollo%20Social/mecanismos_focalizacion_1.pdf

e Busso G. Vulnerabilidad social: nociones e implicancias de políticas para Latinoamérica a inicios del siglo XXI. In: Seminario Internacional: las diferentes expresiones de la vulnerabilidad social en América Latina y el Caribe; 20 a 21 jun 2001; Santiago de Chile. Santiago de Chile: CEPAL, CELADE; 2001 [cited 2015 Mar 13]. Available from: http://www.cepal.org/publicaciones/xml/3/8283/GBusso.pdf

f Ramírez Guerrero J. Capacitación laboral para el sector informal en Colombia: informe presentado a la Oficina Internacional del Trabajo: documento de trabajo. Ginebra: OIT; 2002 [cited 2017 Mar 29]. Available from: http://www.ilo.org/wcmsp5/groups/public/---ed_emp/---ifp_skills/documents/publication/wcms_103989.pdf

g Ministerio del Trabajo (COL); Organización Iberoamericana de Seguridad Social (OISS). II Encuesta Nacional de Condiciones de Seguridad y Salud en el Trabajo en el Sistema General de Riesgos Laborales de Colombia. Bogotá (DC); 2013 [cited 2014 Sep 2]. Available from: http://ccs.org.co/salaprensa/images/Documentos/INFORME_EJECUTIVO_II%20ENCSST.pdf

h Departamento Administrativo Nacional de Estadística (COL). Medición del empleo informal y seguridad social: trimestre móvil febrero - abril de 2013: resúmen ejecutivo. Bogotá (DC); 2013 [cited 2015 Aug 30]. Available from: http://www.dane.gov.co/files/investigaciones/boletines/ech/ech_informalidad/re_ech_informalidad_feb_abr2013.pdf

i Climent IA. Tercer mundo, desarrollo, desastres y tecnología: una mirada desde la geografía. *Serie Geogr* . 2002 [cited 2015 Mar 13];(10):11-26. Disponible en: http://dspace.uah.es/dspace/bitstream/handle/10017/1157/OK-01-Revista-Articulo-Ignacio%2520Alonso%2520-11%2520a%252026%5b1%5d.pdf?sequence=1&isAllowed=y

j Rodríguezoli Vignol J. Vulnerabilidad demográfica: una faceta de las desventajas sociales. Santiago de Chile; CEPAL, ECLAC; 2000 [cited 2015 Mar 14]. (CEPAL- Población y Desarrollo, 5). Available from: http://repositorio.cepal.org/bitstream/handle/11362/7185/S2000937_es.pdf?sequence=1

k Pizarro R. La vulnerabilidad social y sus desafíos: una mirada desde América Latina. Santiago de Chile: CEPAL, ECLAC; 2001 [cited 2015 Mar 1]. (CEPAL- Estudios Estadísticos y Prospectivos, 6). Available from: http://repositorio.cepal.org/bitstream/handle/11362/4762/S0102116_es.pdf?sequence=1

l Kaztman R, Filgueira CH. Marco conceptual sobre activos, vulnerabilidad y estructura de oportunidades. Montevideo: CEPAL, Oficina de Montevideo; 1999 [cited 2015 Mar 14]. Available from: http://www.cepal.org/publicaciones/xml/6/10816/LC-R176.pdf

m Kaztman R. Notas sobre la medición de la vulnerabilidad social. Montevideo: Universidad Católica de Uruguay, Programa IPES; 2000 [cited 2015 Mar 14]. (Serie Documentos de Trabajo del IPES – Colección Aportes Conceptuales, 2). p.76-301. Available from: http://ucu.edu.uy/sites/default/files/facultad/dcsp/vulnerabilidad_social.pdf

n Filgueira CH. Estructura de oportunidades y vulnerabilidad social, aproximaciones conceptuales recientes. Santiago de Chile: CEPAL, CELADE; 2001 [cited 2015 Mar 14]. Available from: http://www.cepal.org/publicaciones/xml/3/8283/cfilgueira.pdf

o Kaztman R, Coordinador. Programa de las Naciones Unidas para el Desarrollo PNUD - Uruguay. Comisión Económica para América Latina y el Caribe CEPAL - Oficina de Montevideo. Activos y estructuras de oportunidades - estudios sobre las raíces de la vulnerabilidad social en Uruguay. Montevideo: PNUD, CEPAL; 1999 [cited 2015 Mar 14]. Available from: http://www.cepal.org/publicaciones/xml/2/10772/caratula.pdf

p Programa de las Naciones Unidas para el Desarrollo (PNUD). Informe sobre Desarrollo Humano 2014: sostener el progreso humano: reducir vulnerabilidades y construir resiliencia: resumen. New York; 2014 [cited 2015 Aug 29]. Available from: http://hdr.undp.org/sites/default/files/hdr14-summary-es.pdf

q Organización Internacional del Trabajo. Aumento del empleo vulnerable y la pobreza: entrevista con el jefe de la unidad de las tendencias mundiales del empleo de la OIT. Ginebra: OIT; 2010 [cited 2013 Mar 5]. Available from: http://ilo.org/global/about-the-ilo/newsroom/features/WCMS_120473/lang--es/ index.htm

r Neffa JC. Sector informal, precariedad, trabajo no registrado. In: 9º Congreso Nacional de Estudios del Trabajo. Grupo 8: Cambios en el trabajo: reestructuración, tercerización y deslocalización de la producción. Buenos Aires: ASET; 2009 [cited 2015 Oct 3]. Available from: http://www.aset.org.ar/congresos/9/Ponencias/p8_Neffa.pdf

s Guataqui Ros RC, García-Suaza A, Rodríguez-Acosta M. El perfil de la informalidad laboral en Colombia. Bogotá: Universidad del Rosario, Facultad de Economía; 2011[cited 2015 Oct 3]. (Documentos de Trabajo, 95). Available from: http://www.urosario.edu.co/urosario_files/84/84048d18-e754-4f80-afc5-f80d4b092260.pdf

t Organización Internacional del Trabajo Resolución sobre la actualización de la Clasificación Internacional Uniforme de Ocupaciones. Estructura de la Clasificación Internacional Uniforme de Ocupaciones. Ginebra: OIT: 2008 [cited 2015 Sep 27]. Available from: http://www.ilo.org/public/spanish/bureau/stat/isco/docs/resol08.pdf
